# Towards Fully Autonomous UAVs: A Survey

**DOI:** 10.3390/s21186223

**Published:** 2021-09-16

**Authors:** Taha Elmokadem, Andrey V. Savkin

**Affiliations:** School of Electrical Engineering and Telecommunications, The University of New South Wales, Sydney 2052, Australia; t.elmokadem@unsw.edu.au

**Keywords:** UAVs, aerial drones, flying robots, autonomous navigation, trajectory planning, obstacle avoidance, intelligent control, collaborative robots’ control, aerial environmental monitoring, UAVs for smart agriculture

## Abstract

Unmanned Aerial Vehicles have undergone rapid developments in recent decades. This has made them very popular for various military and civilian applications allowing us to reach places that were previously hard to reach in addition to saving time and lives. A highly desirable direction when developing unmanned aerial vehicles is towards achieving fully autonomous missions and performing their dedicated tasks with minimum human interaction. Thus, this paper provides a survey of some of the recent developments in the field of unmanned aerial vehicles related to safe autonomous navigation, which is a very critical component in the whole system. A great part of this paper focus on advanced methods capable of producing three-dimensional avoidance maneuvers and safe trajectories. Research challenges related to unmanned aerial vehicle development are also highlighted.

## 1. Introduction

Unmanned Aerial Vehicles (UAVs) have evolved greatly over recent decades with prevalent use in military and civilian applications such as search and rescue [1], wireless sensor networks and the Internet of Things (IoT) [2,3], remote sensing [4], surveillance and monitoring [5,6,7], 3D mapping [8], object grasping and aerial manipulation [9,10], underground mine exploration and tunnel inspection [11,12], etc. Challenges in developing UAVs keep increasing as the complexity of their tasks increases especially with the aim of moving towards fully autonomous operation (i.e., with minimum human interaction). Moreover, many applications require UAVs to autonomously operate in unknown and dynamic environments where they need to completely rely on onboard sensors to understand the environment they navigate in and to complete their tasks efficiently. The autonomous navigation problem can generally be defined as the vehicle’s ability to reach a goal location while avoiding collisions with surroundings without human interaction. This is a very challenging problem as it is important to achieve safe navigation to avoid causing damage or injuries. Limitations on available technologies related to UAVs add more complexities to the development of autonomous navigation methods in order to ensure reliability and robustness compared with unmanned ground vehicles (UGVs) and autonomous underwater vehicles (AUVs). Examples of such are limitations on sensing capabilities, allowed payload capacity, flight time, energy consumption, communication, actuation and control effort. Developing efficient and advanced motion control methods plays a critical role in minimizing the effect of these factors. For example, adopting complex bio-inspired flying behaviors such as perching and maneuvering on surfaces can help extend mission flight time [13].

Many researchers have contributed towards addressing the navigation problem for UAVs. This overview aims at surveying the developments made in the past ten years towards achieving fully autonomous operations. Some key approaches developed earlier than the considered time frame are also reported for the sake of completion. General definitions and research areas are also provided for new researchers interested in this field. Additionally, a list of useful open-source projects and tools is provided which may aid in quick development and deployment of new approaches related to UAVs as part of a complete autonomous stack.

This survey is dedicated to the more complex problem of three-dimensional (3D) obstacle avoidance utilizing the full maneuvering capabilities of UAVs. Given the fact that many of the existing algorithms are developed considering general 3D kinematic models, they are applicable to vehicles moving in 3D, including different UAV types and autonomous underwater vehicles (AUVs). Similarly, some of the general approaches developed for AUVs are also reported here given that they are applicable to UAVs. Planar approaches usually consider flights at a fixed altitude to simplify the obstacle avoidance problem. These approaches may fail with the increased complexity of the environments where UAVs are needed; hence, utilizing 3D avoidance maneuvers is more desirable. However, some planar approaches are also reported here where they can potentially inspire extensions to more general 3D methods.

This paper is organized as follows. A general overview of existing UAV types, classifications, autonomous navigation paradigms, and control structures is given in Section 2. Next, different motion planning and obstacle avoidance techniques are surveyed in Section 3. After that, Section 4 presents different control methods used for UAVs along with information about adopted dynamical models for different UAV types. Brief information about existing localization and mapping techniques is also provided in Section 5. Additionally, some useful open-source projects and tools for UAV development are provided in Section 7. Research challenges are then outlined in Section 8 along with some example applications where UAVs are used. Finally, concluding remarks are made in Section 9.

## 2. UAV Types, Autonomy and System Architectures

### 2.1. UAV Types

UAVs can be classified based on several factors such as size, mean takeoff weight, control configuration, autonomy level, etc. For example, classifications of UAVs based on size according to the Australian Civil Aviation Safety Authority (CASA) are:

- Micro: less than 250 g;
- Very Small: 0.25–2 kg;
- Small: 2–25 kg;
- Medium: 25–150 kg;
- Large: More than 150 kg.

**Figure 1:**
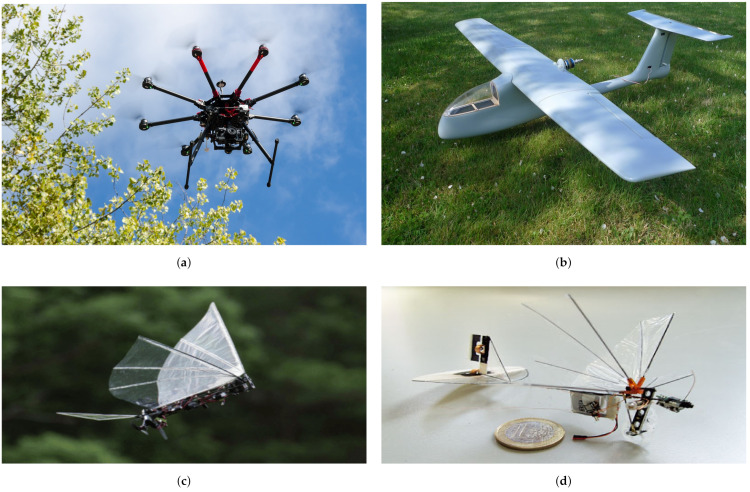
Different UAV types based on control configurations. (**a**) Multirotor (Hexacopter), (**b**) Fixed-Wing, (**c**) Ornithopter flapping-wing UAV (Robo Raven) [37], (**d**) Entomopter flapping-wing UAV (DelFly Micro).

Large UAVs are mainly used in tactical missions and military applications; for more detailed classifications related to military use, see [14]. Based on control configurations, UAVs can be categorized into (see Figure 1):

- single-rotor [15,16,17,18]: helicopter;
- multi-rotor [19,20,21,22,23,24]: tricopter, quadrotor, hexacopter, etc.;
- fixed-wing [25,26,27];
- hybrid [28,29,30,31];
- flapping wings [32,33,34,35,36,37,38]: Ornithopters and Entomopters.

Single-rotor aerial vehicles such as helicopters have not been utilized much as UAV platforms. Multi-rotors on the other hand have become the most popular choice in most civilian applications when it comes to maneuverability. Multi-rotors such as quadrotors, hexacopters and octocopters with fixed-pitch rotors share similar dynamical models for control. However, quadrotors are cheaper, faster, and highly maneuverable while hexacopters and octocopters can offer better flight stability, fault-tolerance, and more payload capacity. Multi-rotors with fixed-pitch rotors are underactuated systems where it is not possible to completely control all degrees of freedom. There have been recent advances in developing omnidirectional tilt-rotor UAVs which are fully actuated in 6DOF [23,24,39,40].

Multi-rotors in general lie under the category of vertical-takeoff-and-landing (VTOL) vehicles with the ability to hover in place. On the contrary, fixed-wing UAVs are horizontal-takeoff-and-landing (HTOL) vehicles, and they cannot hover at a certain position due to nonholonomic constraints. Instead, they have to loiter around areas of interest. However, fixed-wing UAVs have advantages such as long endurance (i.e., flight time) and higher achievable speeds compared to multi-rotors. Hybrid UAVs combine both configurations of fixed wings and multiple rotors utilizing the advantages of both such as vertical takeoff and landing, hovering and long endurance flights. However, these vehicles are still under development, and more research is needed for reliable control, especially when switching between flight modes.

Another type of UAV is one with flapping wings inspired by birds (Ornithopters) and insects (Entomopters). This type is still under development due to its complex dynamics and anticipated power problems [32]. Recently, new bio-inspired hybrid unmanned vehicles have also been proposed to handle navigation in different domains such as underwater-aerial vehicles [41,42,43] and aerial-ground vehicles [44,45,46,47,48].

### 2.2. Autonomy Levels

Being completely able to carry out missions/tasks with minimum human interaction is an ultimate goal for unmanned aerial vehicles. Different levels of autonomy can be achieved towards that goal depending on the complexity of tasks and whether a fully autonomous solution exists or not for that specific application. These levels can be described based on the UAV mode of operation according to the National Institute of Standards and Technology (NIST) as follows [49]:

- **Fully autonomous:** UAV can carry out a delegated task/mission without human interaction where all decisions are made onboard based on sensors observations adapting to operational and environmental changes.
- **Semi-autonomous:** A human operator is needed for high-level mission planning and for interaction during the movement when some decisions are needed that the UAV is not capable of making. The vehicle can maintain autonomous operation in between these interactions. For example, an operator can provide a list of waypoints to guide the vehicle where it can manage to move safely towards these positions with obstacle avoidance capability.
- **Teleoperated:** The remote operator relies on feedback from onboard sensors to move the vehicle either by directly sending control commands or intermediate goals with no obstacle avoidance capabilities. This mode can be used in Beyond-Line-of-Sight (BLOS) applications.
- **Remotely controlled:** A remote pilot is needed to manually control the UAV without sensors feedback which can be used in Line-of-Sight (LOS) applications.

### 2.3. Towards Fully Autonomous Operations

Developing a fully autonomous UAV is a very challenging and complex problem. A modular approach for both hardware and software architectural design is commonly adopted in the literature by most existing autonomous UAVs for a simpler and fault-tolerant solution.

At the hardware level, a UAV in its simplest form consists of a frame, a propulsion system and a Flight Control System (FCS). The UAV’s size and propulsion system can be designed to support the needed payload and flight time as per the mission requirements. A propulsion system consists of a power source (ex. batteries, fuel cells, micro-diesels and/or micro gas turbines), motors drivers or electronic speed controllers (ESCs), motors (ex. brushless DC motors), propellers and/or control surfaces (ailerons, flaps, elevators, and rudders).

The flight control system is simply an embedded system consisting of the autopilot, avionics and other hardware directly related to flight control [14]. For example, main sensors critical to flight control include inertial measurement units (IMUs), barometers/altimeters, and GNSS (for outdoor use). Existing commercial products offer complete systems combining these sensors, which are known as Attitude Heading Reference Systems (AHRSs). More advanced solutions include onboard Kalman filtering to fuse data from all sensors to provide absolute positioning solutions; these are referred to as Inertial Navigation Systems (INSs). The next component is the computing unit (ex. a microcontroller), which is usually used to implement the autopilot logic for reliable and fault-tolerant flight control. Ideally, the computing unit must be subject to real-time constraints. That is, its response must be deterministic and within specified time constraints. In general, FCS is responsible for computing low-level control commands, estimating the vehicles states (altitude, attitude, velocity, etc.) based on sensor data, logging critical information for post-flight analysis, and interfacing with higher level components either by wired connection or through other communication links. Having a FCS is enough to allow teleoperation navigation mode where a remote operator can directly send waypoints and/or control commands. It is also possible to achieve semi-autonomous operations in simple environments where reactive control methods with low computational cost are implemented within the autopilot to provide basic collision avoidance capabilities.

For more complex tasks/missions, an onboard computer with higher processing power, namely a mission computer, is required to achieve fully autonomous operations given that a UAV with proper size and power is used. In this structure, the mission computer usually implements high-level mission and motion planning by relying on information interpreted from high-bandwidth sensory data in addition to running required processes with expensive computational cost. It can also have its own communication link with a Ground Control Station (GCS) to stream high-bandwidth data such as images and depth point clouds.

**Figure 2:**
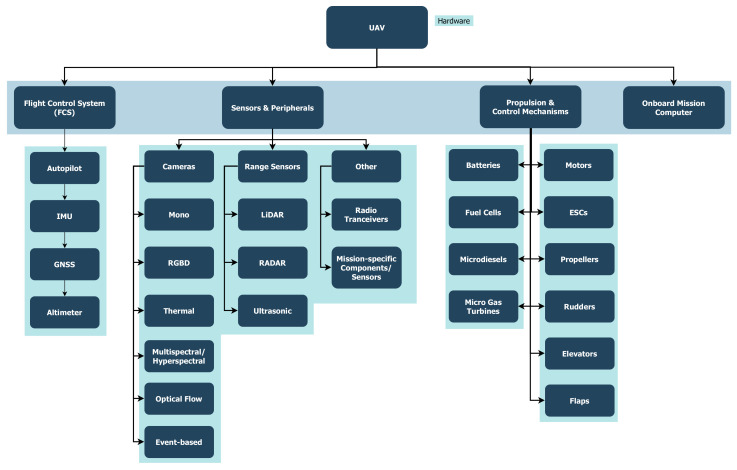
System architecture showing hardware components commonly used with UAVs.

**Figure 3:**
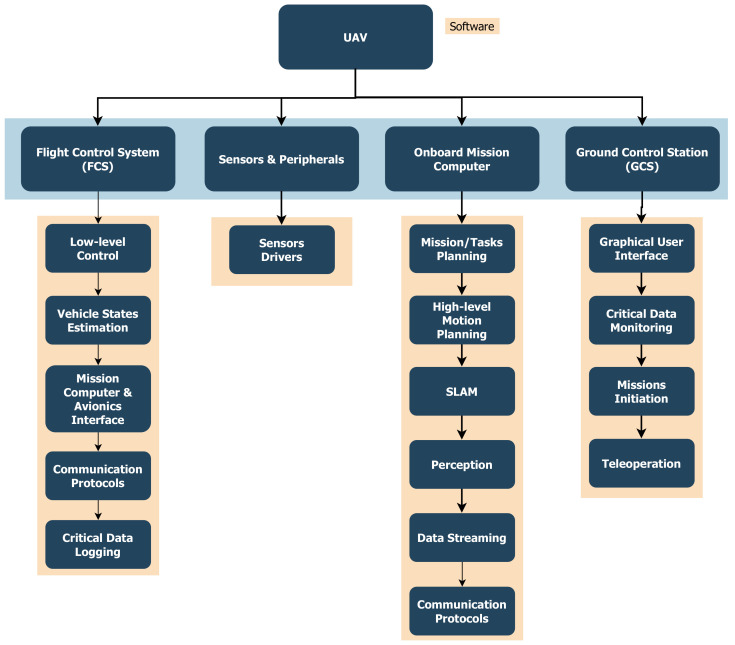
System architecture showing software components commonly used with UAVs.

**Figure 4:**
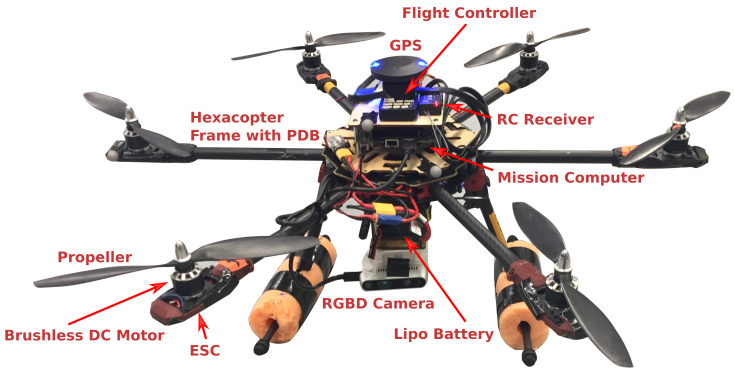
Example UAV setup of a hexacopter UAV type with FCS, mission computer and an RGBD sensor.

Different kinds of sensors can be used for advanced perception and planning, depending on the mission requirements, UAV available payload and power, and environmental conditions. Examples of commonly used sensors are cameras (monocular, RGBD, thermal, hyperspectral, etc.), range sensors (LiDAR, RADAR, ultrasonic) and other task-specific sensors (ex. grippers, manipulators, sprayers, etc.). A summary of hardware and software components used with UAVs are shown in Figure 2 and Figure 3, and an example hexacopter is presented in Figure 4 showing the system components for some use case.

The software architecture of the autonomous stack implemented on the mission computer typically consists of several processes/modules running in parallel and a messaging middleware is used to interchange messages between processes on the mission computer or with other computers on the same network (for example, in multi-UAV systems). Some of these modules are related to the mobility aspects that can ensure safe navigation which can be common among most UAV systems and other autonomous mobile robots. Other modules would implement logic that is application-specific such that the UAV can autonomously perform the delegated task. For example, in fire-fighting applications, a UAV is needed to autonomously locate and extinguish fires, which requires additional modules to be included within the autonomous stack including computer vision pipelines and an extinguisher control mechanism. In many remote sensing applications, the main task could be only collecting data either in the form of images or information from other onboard sensors to be analyzed and processed post-flight.

**Figure 5:**
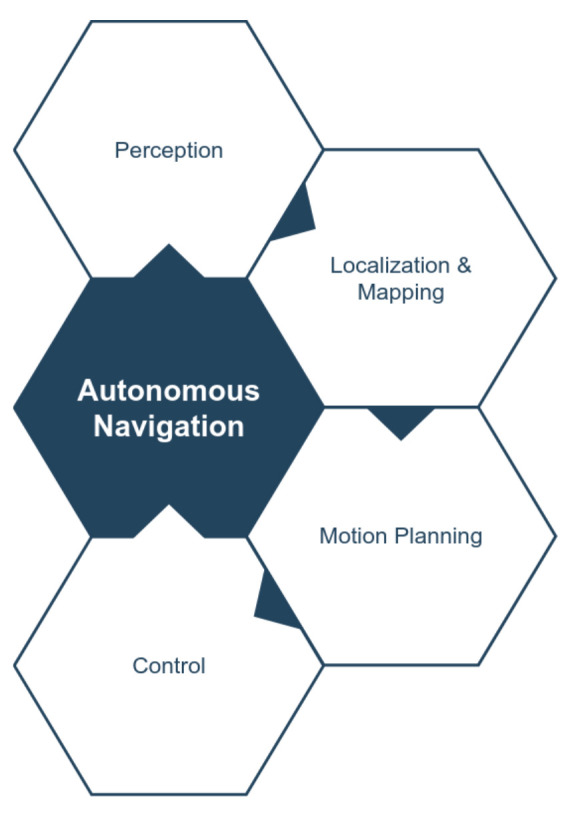
Modular software structure for UAV navigation stack.

Mobility-related modules are the core components needed to ensure collision-free navigation in all applications. By considering only the mobility-related components, a popular modular structure for autonomous navigation is adopted in the literature which consists of the following modules/subsystems (Figure 5):

- Perception;
- Localization and Mapping;
- Motion Planning and Obstacle Avoidance;
- Control.

This modular approach of addressing the navigation problem offers a flexible expandable design with fault-tolerance. However, other possible designs can also be seen for less complex tasks or for vehicles with very limited resources by coupling control and planning, without the need for localization and mapping, in a reactive fashion, as will be shown in the next section.

## 3. Navigation Techniques

A crucial part of autonomous navigation is to ensure that the vehicle can move while avoiding collisions with its surroundings. This is a general problem in robotics which can be addressed by motion planning or reactive control. Generally, the motion planning problem can roughly be described as trying to find collision-free trajectories between initial and final *configurations* while satisfying some kinematic and dynamic constraints. A *configuration* in this case refers to the position and orientation of a mobile robot where a *configuration space* is the set of all possible configurations. The dimension of the configuration space equals the number of controllable degrees of freedom. For example, planning motions for quadrotors can be carried out in a space of their 3D position coordinates and heading (yaw) angle while motions for omnidirectional (fully actuated) UAVs can be planned considering all translational and rotational states (6DOF).

In a decoupled approach, the UAV control system can execute motions planned by a high-level system, namely a motion planner, where these plans need to be feasible and safe (i.e., collision-free). In other implementations, motion planning can be coupled with the control system design where reactive control laws are developed to directly generate obstacle avoidance maneuvers based on sensor measurements. Some refer to those in the literature in loose terms as obstacle/collision avoidance methods. The term collision avoidance is mostly used by the UAV research society in referring to avoiding collisions with other cooperative or noncooperative aerial vehicles (i.e., dynamic obstacles) sharing the same flight space, while the term obstacle avoidance may be used more often in indoor, industrial and urban environments where the flight space is filled with other static/dynamic obstacles. That is, high-altitude flights commonly adopt the collision avoidance terminology and low-altitude flights may use the more general obstacle avoidance term. This terminology is also adopted more often in multi-UAV systems to differentiate between methods that only consider collision avoidance among the vehicles within the system to those that also consider obstacle avoidance in obstacle-filled environments.

### 3.1. Navigation Paradigms

**Figure 6:**
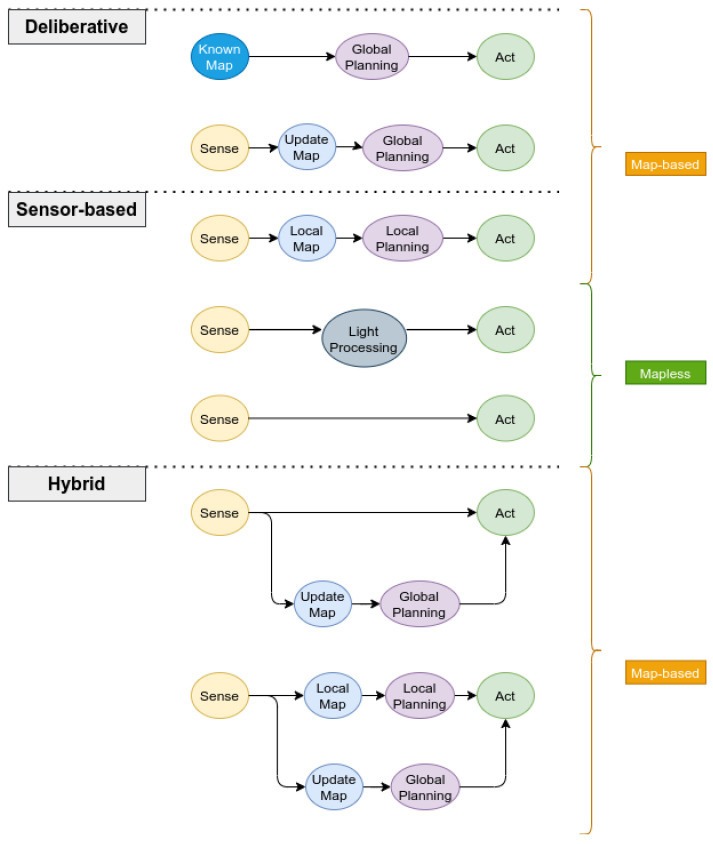
Different paradigms adopted for autonomous navigation from a high-level perspective.

Existing navigation techniques for autonomous mobile robots in general can be classified into *deliberative* (global planning), *sensor-based* (local planning) or *hybrid* (see Figure 6). *Deliberative* approaches require a complete knowledge of the environment represented as a map. Global path planning methods can then be used to search for safe and optimal paths. Classical path planning algorithms can be categorized into:

- Search-based methods (ex. Dijkstra, $A^{*}$, $D^{*}$, etc.);
- Potential field methods (ex. navigation function, wavefront planner, etc.);
- Geometric methods (ex. cell decomposition, generalized Voronoi diagrams, visibility graphs, etc.);
- Sampling-based methods (ex. PRM, RRT, RRT*, FMT, BIT, etc.);
- Optimization-based methods (PSO, genetic algorithms, etc.).

Many of these methods can find optimal paths if one exists at the expense of requiring full knowledge about the environment which is not suitable in unknown and dynamic environments. For more detailed information about such planning methods, the reader is referred to [50].

On the other hand, *sensor-based* methods rely directly on current sensor measurements or a short history of the sensors’ observations (i.e., a local map) to plan safe paths in real time. The planning horizon can typically be very short for some period ahead of time or it could be carried out at each control update cycle in a receding horizon fashion. A special class of such methods is made up of reactive approaches where sensor measurements are coupled to control actions either directly [51] or after light processing [52]. Sensor-based methods offer solutions with great computational performance which makes them favorable for navigation problems in unknown and dynamic environments. These methods do not generate global optimal solutions as they do not use complete knowledge about the environment during motion; however, it is possible to find locally optimal solutions. In practice, it is common to sacrifice optimality for computation speed, especially when considering micro-UAVs with fast dynamics and limited computing power. Sensor-based methods are also prone to getting stuck sometimes due to the local minimum.

*Hybrid* approaches combine both deliberative and sensor-based methods to generate a more advanced navigation behavior benefiting from the advantages of both classes. It relies on low-latency local planning or reactive control to handle unknown and dynamic obstacles while using a high-level global planning method to guide the vehicle utilizing accumulated knowledge about the environment.

### 3.2. Map-Based vs. Mapless Methods

Navigation methods can alternatively be classified into *map-based* or *mapless* approaches [53,54]. This classification highlights the computational complexity including memory requirements and whether they rely on accurate localization and mapping or not.

*Map-based* strategies require a local (or global) map representation of the environment which can be provided before navigation starts (deliberative approaches) or it can be built during navigation based on sensor measurements (some sensor-based approaches). Safe paths can then be found using local/global planning algorithms based on either *metric* or *topological* maps. Therefore, such methods are demanding in terms of computational resources, planning time and memory requirements, which is highly dependent on the environment size and its complexity. Nevertheless, local map-based methods are very commonly used with UAVs to generate local optimal solutions for technological advances where it is possible to have mini lightweight computers with high processing power onboard.

On the contrary, *mapless* strategies (reactive methods) rely directly on sensor measurements to make motion decisions without the need for maintaining global maps and accurate localization (except when using GNSS). Hence, control actions can be directly coupled with either visual clues from image segmentation, optical flow or feature tracking in subsequent frames in vision-based methods [54] or interpreted information from range sensors and 3D point clouds such as relative distance to obstacles, gaps or bounding objects. These methods offer the best computational complexity for obstacle avoidance as control is coupled with planning through light processing of sensor data which can provide very quick reflex-like reactions to obstacles. Some of the challenges when developing purely reactive navigation methods is the possibility of getting stuck in local minimums, and a limited field of view (FOV) may affect the overall performance. Additionally, fast reactions to obstacles achieved by reactive methods come at the cost of generating nonoptimal solutions in some cases due to the fact that they do not utilize information about previously sensed obstacles.

### 3.3. Overall Navigation Control Structure

**Figure 7:**
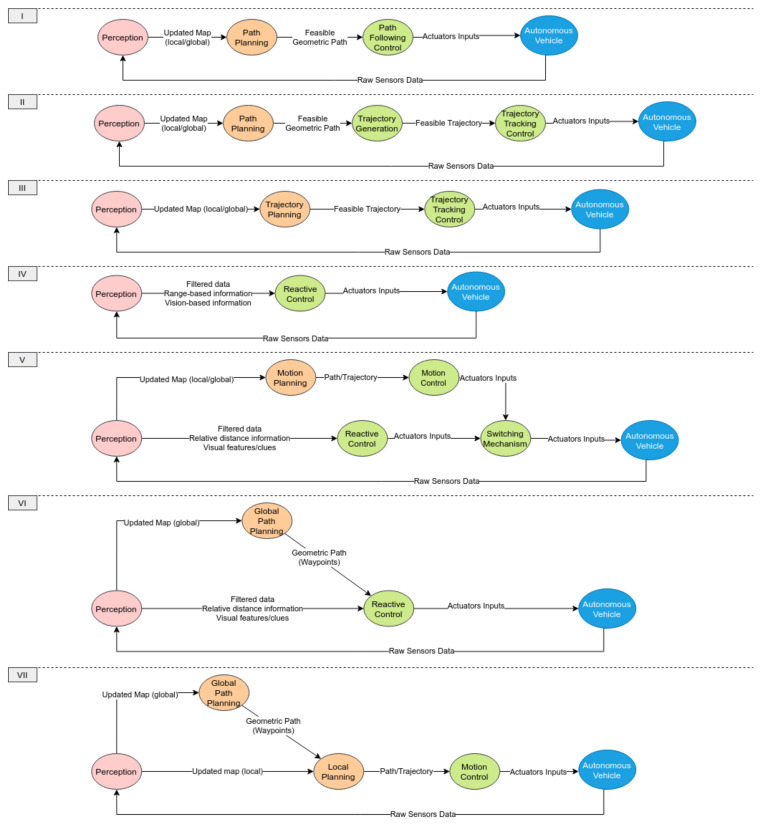
Different autonomous navigation control structures.

From a control perspective, different structures were adopted in the literature to deal with the high complexity of the navigation problem. As mentioned before, the most common structure is based on decoupling planning and control due to its simplicity in design. One can categorize the existing methods into seven different control structures, as shown in Figure 7. Structures I–III show the general decoupled approach where motion planning and control are decoupled, while structure IV is used by reactive approaches which directly couple planning and control. Structures V–VII correspond to hybrid approaches which can be a combination of structures I–IV.

In decoupled approaches, some motion planning methods further simplify the problem by subdividing it into two stages. The first stage simply tries to find a collision-free geometric path satisfying kinematic constraints. Constraints can be considered directly in the planning algorithm, or the entire process can be further decomposed into finding a safe path first ignoring such constraints then applying path smoothing techniques to satisfy the kinematic constraints. Then, it is followed by a trajectory generation stage to obtain feasible trajectories satisfying dynamic constraints. Other approaches tackled this problem by directly planning trajectories using optimization-based methods which is a harder problem to solve.

To differentiate between different motion planning paradigms, we highlight the differences between *path planning* and *trajectory planning/generation*. *Path planning* is the process of finding a geometric collision-free path between starting and end positions without a timing law. In *trajectory planning*, a timing law is associated with the planned collision-free geometric path represented as a *trajectory* which includes information about higher derivatives (i.e., velocity, acceleration, etc.). Trajectories are mostly planned to satisfy dynamic constraints which can then be passed to a control system adopting a trajectory tracking control design. One of the common approaches for trajectory planning is by using a path planning algorithm to find an initial geometric path followed by formulating trajectory generation as an optimization problem to plan local optimal trajectories around the initial path subject to several constraints. Alternatively, some approaches adopt ideas from missile guidance, implementing path following control laws to track the geometric path given that it satisfies nonholonomic constraints whenever they exist without generating a trajectory.

In the following subsections we will survey recent works adopting local motion planning or reactive paradigms in accordance with the considered control structures.

### 3.4. Local Path Planning

A number of existing methods treat the problem through applying path planning algorithms locally to find feasible geometric paths assuming a general 2D/3D kinematic model. Examples of these methods include sampling-based [55,56,57,58], graph-based [59,60] and optimization-based methods [61,62,63]. These methods are developed at a high level considering only kinematic constraints assuming a low-level path following controller exists to execute the planned paths while satisfying the dynamic constraints similar to control structure I. They can also be combined with a trajectory generation method similar to structure II.

Adopting sampling-based methods helps address the high dimensionality problem of the 3D search space to generate collision-free paths in real time, which was considered in many works. In [56], a planning algorithm was proposed for rotary-wing UAVs. It decouples the motion planning problem into two stages, namely path planning and path smoothing, which is a common approach to simplify the problem, especially when nonholonomic constraints need to be satisfied (ex. for fixed-wing UAVs); for example, see [64,65]. A sampling-based planning algorithm, namely RRR, was adopted to search for collision-free paths followed by a path smoothing algorithm such that the smoothed path can satisfy curvature continuity and nonholonomic constraints. An analytical solution for the adopted path smoothing algorithm was also presented in [66] considering smoothing of 3D paths. An explicit path-following model predictive control (MPC) was used in [56] to ensure that the vehicle can track the planned paths, and it was formulated based on a linear model of the motion with no constraints. Another real-time path planning algorithm was suggested in [55] based on chance-constrained rapidly exploring random trees (CC-RRT) for safe navigation in 2D constrained and dynamic environments. The motion planning relies on a proposed clustering-based trajectory prediction to model and predict future behavior of dynamic obstacles. This motion prediction algorithm combines Gaussian processes (GPs) with the sampling-based algorithm RRT-Reach to cope with GP shortcomings such as the high computational cost. Another RRT variant, namely Closed-Loop RRT, was used in [57] to handle navigation in 3D dynamic environments. In [58], a sampling-based approach was adopted in an informative path planning framework where the goal is to generate safe paths that can maximize the information gathered during movement, which is important in exploring unknown environments.

Some other works formulated the 3D path planning problem as an optimal control problem, such as [61,62]. The authors of [61] formulated the optimal control problem in 2D to satisfy time and risk constraints as the 3D optimal control problem would be harder to solve. Then, a 3D path was approximated in a final stage based on a terrain height map. In contrast, the method in [62] presented a path planner based on a 3D optimal control problem formulation where a model based on artificial potential field (APF) was used. Other optimization-based methods considered parallel genetic algorithm and particle swarm optimization as in [63].

### 3.5. Local Trajectory Planning

A more popular approach in addressing the local planning problem for UAVs is through planning feasible trajectories to further satisfy dynamical constraints and optimality of path smoothness with respect to higher derivatives enabling high-speed and aggressive movements. Generating smooth trajectories is important for high-speed applications to avoid sudden changes in actuators’ accelerations and mechanical vibration problems [67]. Therefore, it can be seen from the literature that control structures II–III are commonly used for aggressive maneuvers, whether by combining path planning and trajectory generation, as in [59,68,69,70,71,72,73,74,75,76], or by direct trajectory planning, as in [77,78,79,80,81,82,83,84,85,86].

Generally, many of these approaches represent the trajectories as piecewise polynomials where the polynomial coefficients are used as decision variables in the optimization problem. Some works use Bernstein and B-splines polynomial bases and utilize their properties when formulating the problem [76,78,87]. For example, Bezier curves are known for their convex hull property, where a whole trajectory segment can be contained within a convex region by adding constraints on its Bezier control points.

A trajectory generation method for quadrotors was suggested in [68] to find minimum-snap trajectories between specified keyframes provided by a high-level planner with corridor-like constraints, representing the convex decomposition of free space. This pioneering approach was adopted in several studies such as [59,70,72,73,88]. The work [70] formulated the trajectory generation as a mixed-integer optimization problem to generate minimum-jerk polynomial trajectories constrained to convex collision-free regions with other constraints on velocity and acceleration. The authors have also proposed a way to generate the safe convex regions using Iterative Regional Inflation by Semi-definite programming (IRIS), which was initially proposed in [89].

Similarly, a real-time trajectory generation method was proposed in [59] for quadrotors, presenting another way of determining such safe convex regions. It relies on online built voxel maps and short-range planning algorithm where it uses an $A^{*}$ search method to find a safe path in a discretized graph representation of the voxel map. The path is then inflated to generate a set of connected polyhedrons specifying the collision-free regions around the path, resulting in corridor-like constraints. This approach was further developed in [72] to provide a more robust and efficient solution which was implemented in [88], showing a complete system for autonomous flights of multi-rotors in GPS-denied indoors environments. A minimum-jerk trajectory is then computed similar to the approach in [73] where a convex optimization problem is formulated by confining the trajectory spline segments to be within specified flight corridors with constraints to ensure the continuity of the trajectory splines. This approach avoids the more complex nonconvex problem formulation that results when considering the trajectory planning problem with constraints corresponding to collisions with obstacles.

The works [72,73] adopt a receding horizon planning paradigm to plan trajectories over finite time intervals with safe stopping policies in case of planning failure. The works [59,73] adopt a short-range planning paradigm where a set of candidate goals within the current sensing FOV are used for trajectory planning until the global goal is reached. In contrast to expressing collision-free constraints as convex decomposition of free space, the authors of [79] suggested a different approach to efficiently handle dynamic and cluttered environments as the authors claimed that the conventional convex decomposition of free space can be conservative and may become harder in dynamic environments. This approach is based on using planes to represent the separation between the polyhedral representations of each trajectory segment. Additionally, the authors use decision variables to represent these planes within the optimization problem.

Another optimization-based method was suggested in [69] as an extension to [68] by formulating the minimum-snap trajectory generation problem as an unconstrained quadratic program (QP). This trajectory generation can be combined with a 3D kinematic planner to generate safe geometric paths where the authors have considered the RRT* planner in their implementation. Additional iterative steps are needed if the generated trajectories were found in collision where the optimization problem is repeatedly resolved using safe intermediate waypoints until a collision-free trajectory is obtained.

In contrast to optimization-based trajectory generation where dynamic constraints are considered in the optimization problem, motion primitives were considered as a simpler computationally efficient way to generate collision-free trajectories in 3D in some works such as [77,90,91,92,93,94]. Motion primitives offer a light-weight algebraic solution to the problem which can then be checked for dynamic constraints violation. The low-computational cost of such methods allows for high-speed and aggressive movements since it is possible to quickly search over a large number of motion primitives to achieve a certain goal [90]. Motion and sensing uncertainty were also considered in some methods at planning time such as [94].

Generally, considering dynamic constraints and constraints due to collisions with obstacles in the planning problem makes it harder to solve in real time, causing potential convergence problems. This is known as kinodynamic planning, which is a motion planning problem in a higher dimensional space with differential and obstacle constraints [95]. Some approaches, however, have tackled this more complex problem rather than decoupling the path planning and trajectory generation such as [74,75,96]. The work [74] addressed the trajectory planning problem as a 3D Optimal Control Problem (OCP) with soft obstacle avoidance constraints on a nonconvex quadratic optimization problem. To reduce the computational burden of solving the formulated OCP, constraints based on a reduced number of obstacles, the most threatening ones, were considered. In [75], trajectory planning and control of quadrators in constrained environments was achieved through a formulation as a minimum-time optimal control problem with several constraints on states and inputs, and it was based on the full 6DOF dynamical model. The general problem was reformulated using a change of coordinates and state-input constraints relaxation to reduce the high computational complexity of the original constrained problem.

The motion planning problem for multi-rotors among dynamic obstacles was tackled in [96] at the control level using a nonlinear model predictive controller (NMPC) based on a cost function in terms of the tracking error, input cost and input smoothness cost. Addressing path planning using a pure NMPC structure is challenging as it is computationally expensive to solve nonconvex optimization problems in real time. Therefore, [96] considered a new solver for such nonlinear nonconvex problems known as Proximal Averaged Newton for Optimal Control (PANOC) [97,98] to make the solution more appealing. There exists an open-source implementation of this solver which is OpEn (Optimization Engine) [99]. A similar approach was also considered in [100].

Formulating the 3D trajectory planning as a Quadratic Program (QP) was also considered in [71,78,82]. In [71], an optimization-based method was proposed to generate locally optimal safe trajectories for multi-rotor UAVs using high-order polynomial splines. The optimization problem was formulated to minimize costs related to higher order derivatives of the trajectory (ex. snap) and collisions with the environment. The objective function computes collision costs using a Euclidean Signed Distance Field (ESDF) function with a voxel-based 3D local map of the environment. The optimization problem was formulated as an unconstrained quadratic program (QP) so that it can be solved in real-time. The work [78] adopted a mixed-integer quadratic program formulation allowing the solver to choose the trajectory interval allocation, and the time allocation is found by a line search algorithm initialized with a heuristic computed from the previous replanning iteration. Another kinodynamic planner for quadrotors was introduced in [82] using a sampling-based method in combination with an additional optimization-based stage using a sequence of QPs to refine the smoothness and continuity of the obtained trajectory.

Recently, there has also been some growing interest in the field of perception-aware trajectory planning considering perception constraints in the planning problem. The developed methods in this area take into account perception quality to minimize state estimation uncertainty [101], which can be achieved by keeping specific objects/features in the vehicle’s sensing FOV [80]. Examples of such methods can be seen in [80,101,102,103,104,105].

### 3.6. Reactive Methods

Most of the existing reactive methods are developed at a higher level considering different abstractions of UAV 2D/3D kinematic models with velocities/accelerations as control inputs. Collision avoidance can be ensured rigorously for some of these methods under certain technical assumptions [51] in contrast to other motion planning methods. For example, the design may rely on assumptions made about obstacles (shape, size, velocity profile, etc.), environment (static or dynamic) and sensing capabilities (vision-based, distance-based, FOV, range, etc.). Many of the existing reactive methods are planar, which can generally be applied to various types of mobile robots including UAVs moving at a fixed altitude; examples of such methods include [106,107,108,109,110,111,112,113,114]. Adopting these methods for vehicles that can navigate in 3D, such as UAVs and AUVs, becomes less efficient. Therefore, there has been a growing interest in developing 3D reactive navigation methods which will be the main focus in this section in addition to some of the 2D vision-based approaches sufficiently suitable for UAVs in some applications.

A number of geometric-based reactive collision avoidance methods focused on noncooperative scenarios (i.e., dynamic environments) for fixed-wing UAVs or vehicles with nonholonomic constraints adopting the idea of collision cones such as [115,116,117,118,119,120]. Many of these approaches use linear or nonlinear guidance laws to align the velocity vector (i.e., controlling heading and flight path angles) in a certain direction while keeping a constant relative distance to the obstacle to avoid collisions. The work [115] proposed two guidance laws for collision avoidance in static and dynamic environments based on collision cones where the vehicle is guided to track the surface of a safety sphere around the obstacle. Similarly, the works [116,117] adopted collision cones to safely guide fixed-wing UAVs in 3D dynamic environments. In [118], a 3D reactive navigation law was proposed based on relative kinematics between the vehicle and obstacles decoupled into horizontal and vertical planes. Obstacles were modeled as spheres, and collision cones were used for obstacle avoidance. This method was further developed in [119] where a reactive optimal approach was suggested for motion planning in dynamic environments.

A different implementation of collision cones was carried out in [120] for AUVs; however, the same idea can be applied to UAVs as well. No assumptions were made about the obstacle shape; however, obstacles were modeled as spheres for mathematical development, and it was only assumed that the collision cone to the obstacle can be interpreted from sensor measurements. This method relied on maintaining a constant avoidance angle from a nearby obstacle while ensuring a minimum relative distance is achieved. The same problem was addressed differently in [121] where a new nature-inspired 3D obstacle avoidance method for AUVs was developed based on concepts from fluid dynamics.

Another 3D reactive approach was developed in [122,123,124] adopting the idea of avoidance planes with more flexibility in choosing the orientation of these planes while circumnavigating around obstacles.

A different class of 3D reactive methods modified the Velocity Obstacle (VO) approach to allow navigation in dynamic environments such as [125,126]. In [125], the proposed method relied on decoupling the 3D motion to achieve constant relative bearing and elevation in both the horizontal and vertical planes simultaneously. It was assumed that the desired relative bearing and elevation with respect to the noncooperative vehicle can be estimated using onboard cameras. Additionally, the authors of [126] proposed an improvement to the Velocity Obstacle (VO) method to handle 3D static and dynamic environments.

Artificial potential field was also considered in some approaches to handle navigation in dynamic environments as in [127,128,129]. The approaches [127,128] developed modified APF methods for 3D nonholonomic vehicles, while the work [129] designed an APF reactive controller for quadrotors. The approach in [129] combines obstacle avoidance control law based on artificial potential field with a trajectory tracking control law using on a null-space-based scheme at the kinematic level where the obstacle avoidance input has the higher priority. A dynamic controller was then proposed to generate low-level input to ensure that velocities generated by the kinematic controller can be tracked.

The authors of [130] suggested a different 3D navigation approach for rotorcraft UAVs where an escape waypoint is determined whenever an obstacle is detected. Obstacle detection was carried out by extending a cylindrical safety volume from the UAV position along the movement direction in a 3D local map representation of the environment. The escape waypoint is determined by performing a search through a set of concentric ellipsoids around the detected obstacles by iteratively incrementing the ellipses radii until a safe escape point is found. Due to the low complexity of the algorithm, it belongs to the reactive class.

In [131], a computationally light approach was suggested through real-time deformations of a predefined 3D path based on the intersection between two 3D surfaces determined according to the free space and obstacles. Either one or both surfaces are modified in the presence of obstacles such that the intersection between the two surfaces provides a path around the obstacle. To that end, proper functions need to be carefully chosen to represent the obstacle where the authors considered a Gaussian function whose parameters require proper tuning. A path following controller was also proposed based on multi-rotor full dynamical model where a cascaded approach for control was adopted for position and attitude. This was further implemented in [132] where a depth camera was used to detect obstacles. Another 3D reactive method adopting the idea of real-time deformable paths around dynamic obstacles was also proposed in [133].

A number of reactive methods considers vision-based structure such as [83,84,86,134,135]. In [134], a vision-based reactive approach was proposed for quadrotor MAVs based on embedded stereo vision. Obstacles are detected from stereo images-based U-V disparity maps. A short-term local map is built for planning purposes representing approximations of detected obstacles as ellipsoids. Hence, no accurate odometery is needed since no global map is built. The obstacle avoidance algorithm is mainly 2D to find the shortest path along obstacles’ edges. On the other hand, the works [83,84,86] proposed 3D mapless vision-based trajectory planning methods using depth images which can be considered reactive as the planning horizon becomes very short. A different vision-based 3D reactive method was proposed in [135] based on NMPC for quadrotors navigating in dynamic environments.

Some other methods relied on 3D distance measurements (i.e., 3D pointclouds) obtained from LiDAR sensors or depth cameras such as [12,100]. The method proposed in [100] combined 3D collision avoidance with control in a nonlinear model predictive control scheme considering both dynamic and geometric constraints at the same time. It adopted a mapless approach by relying on a subspace clustering method applied to 3D point clouds obtained directly from a 3D LiDAR sensor. On the contrary, a 3D reactive approach was suggested in [12] to allow navigation in tunnel-like environments. Guidance control laws were developed to guide the UAV by directly extracting clues from 3D pointclouds to determine a progressive direction to advance through the tunnel.

Concepts from machine learning were also considered recently in some reactive methods to address obstacle avoidance problems for UAVs. However, these methods are more computationally expensive than other reactive methods, and there are still concerns related to how guaranteed a collision avoidance is as the performance relies on how good the training/learning stage is. Additionally, many of the existing approaches consider only generating motion decisions/policies in 2D without utilizing the full maneuverability of UAVs. Most of these methods are based on deep reinforcement learning [136,137,138,139,140,141,142,143] and deep neural networks [144,145,146,147,148,149,150,151].

A summary of the surveyed local motion planning methods is presented in Table 1.

## 4. UAV Modeling and Control

### 4.1. Modeling

For control design and simulation purposes, it is necessary to have a valid mathematical model that can express the UAV motion. Generally, such a model consists of two main parts which are *kinematics* and *dynamics*. *Kinematic* equations are mainly derived to represent the geometrical aspects of the motion in 3D spaces through defining translation and rotation relationships between different coordinate frames. *Dynamics* can be obtained through the application of Newton laws for a moving rigid body to derive linear and angular momentum equations. Application of Newton laws requires an inertial reference frame I to be defined. On the other hand, analyzing forces and torques acting on the vehicle needs to be carried out with respect to a coordinate frame attached to the moving vehicle (i.e., a body-fixed frame B). Clearly, different UAV types would have some differences in their dynamic equations depending on the actuators’ configurations and other external forces and torques acting on the vehicle. For simplicity, the origin of the body-fixed frame is commonly chosen to coincide with the vehicle’s center of mass. Note that there are other coordinate frames that can be used for different purposes for navigation and control such as Earth-Centered, Geodetic and wind coordinate frames. For more details about these coordinate frames, see [14].

A rotation matrix between the inertial and body-fixed coordinate frames can be used to define the attitude/orientation of the UAV. It is also common to use other representations such as Euler angles (i.e., roll ϕ, pitch θ and yaw ψ) and quaternions $q\in {I\;R}^{4}$. Quaternions are more computationally efficient and do not have the gimbal lock problem while Euler angles are easier to understand physically and can be decoupled into separate degrees of freedom under some assumptions for simplicity.

Let the Euler angle vector be $\Phi ={\left[\phi ,\theta ,\psi \right]}^{T}$, and consider a quaternion vector $q\;=\;{\left[q_{1},q_{2},q_{3},q_{4}\right]}^{T}$. Notice that with Euler angles, usually three rotations are applied in a specific order which can result in different forms for the rotation matrix. The following is an example considering the rotation order ZYX,

(1) $${}_{B}^{I}R\left(\Phi \right)=\left[\begin{array}{ccc} c_{\theta }c_{\psi } & s_{\phi }s_{\theta }c_{\psi }-c_{\phi }s_{\psi } & c_{\phi }s_{\theta }c_{\psi }+s_{\phi }s_{\psi }\\ c_{\theta }s_{\psi } & s_{\phi }s_{\theta }s_{\psi }+c_{\phi }c_{\psi } & c_{\phi }s_{\theta }s_{\psi }-s_{\phi }c_{\psi }\\ -s_{\theta } & s_{\phi }c_{\theta } & c_{\phi }c_{\theta } \end{array}\right]$$

where $c_{\alpha }:=\cos\alpha$, and $s_{\alpha }:=\sin\alpha$. Note that ${}_{B}^{I}R\left(\Phi \right)$ represents the rotation from the body-fixed frame to the inertial frame. Furthermore, ${}_{I}^{B}R\left(\Phi \right)={}_{B}^{I}R^{T}\left(\Phi \right)$.

For a velocity vector expressed in the body-fixed frame, it can be transformed to the inertial frame as follows:

(2) $${}^{I}v={}_{B}^{I}R\left(\Phi \right)\;{}^{B}v$$

such that ${}^{I}v={\left[\dot{x},\dot{y},\dot{z}\right]}^{T}$ and ${}^{B}v={\left[u,v,w\right]}^{T}$. Additionally, the angular velocity can be transformed from B to I as:

(3) $$\dot{\Phi }=T\left(\Phi \right)\Omega$$

where

(4) $$T\left(\Phi \right)=\left[\begin{array}{ccc} 1 & s_{\phi }t_{\theta } & c_{\phi }t_{\theta }\\ 0 & c_{\phi } & -s_{\phi }\\ 0 & s_{\phi }/c_{\theta } & c_{\phi }/c_{\theta } \end{array}\right]$$

with $t_{\theta }:=\tan\theta$. The gimbal lock problem can be seen clearly from T(Φ) where a singularity occurs when $\theta =\pm 90^{o}$. Such a problem does not exist when using quaternions.

Hence, the general model for a UAV is given by:

(5) $$\begin{array}{ccc} \dot{p} & = & {}_{B}^{I}R\left(\Phi \right)\;{}^{B}v \end{array}$$

(6) $$\begin{array}{ccc} {}^{B}\dot{v} & = & \frac{F}{m}-\Omega \times {}^{B}v \end{array}$$

(7) $$\begin{array}{ccc} {}_{B}^{I}\dot{R} & = & {}_{B}^{I}R\Omega \end{array}$$

(8) $$\begin{array}{ccc} \dot{\Omega } & = & I^{-1}\left(M-\omega \times I\Omega \right) \end{array}$$

where $p,{}^{I}v\in {I\;R}^{3}$ are the position and linear velocity expressed in the inertial frame, $\Omega \in {I\;R}^{3}$ is the angular velocity defined in the body-fixed frame, $m\in {I\;R}^{+}$ is the UAV’s mass, and $I\in {I\;R}^{3\times 3}$ is the inertia matrix. Furthermore, $F\in {I\;R}^{3}$ and $M\in {I\;R}^{3}$ correspond to external forces and torques acting on the vehicle.

Modeling the forces and torques differ based on the UAV type, design and actuators configuration which affects the control system design. Example of these differences can be seen in the complete models for fixed-pitch multi-rotors [68,152,153], variable-pitch multi-rotors [23,40], helicopters [17], fixed-wing UAVs [154], flapping-wing UAVs [33], etc. Some researchers have further extended the UAV modeling considered in the control design to include some added systems such as cable-suspended payload [155,156].

### 4.2. Low-Level Control

As mentioned earlier, a common approach to handle the navigation problem is by decoupling planning from control. Thus, a low-level control can be designed independently to track the generated reference paths, trajectories, heading/flight path angles or velocity/acceleration commands. Typically, control laws are developed to minimize tracking errors by determining required input forces and body torques which can then be mapped into motor and actuator commands depending on the UAV type. State estimation is a very critical component for feedback control. Extended Kalman Filter (EKF) is a popular choice in many implementations to provide estimates for the UAV attitude, linear and angular velocities by fusing data from different sensors. Position can also be estimated by fusing information from a positioning source such as GNSS, visual odometry, external positioning system, etc.

A cascaded approach is very common in different control structures where the attitude dynamics (i.e., (7) and (8)) are decoupled to avoid considering the full nonlinear system dynamics in the control design [157]. A high-bandwidth inner loop attitude controller is used to ensure that the vehicle can accurately track reference attitude or angular velocity commands. This reduces the control problem to design an outer control loop for the translational dynamics (5) and (6) that can achieve position/velocity tracking by deciding proper laws in terms of thrust, attitude and/or angular velocities. Several control techniques were adopted in the literature, such as PID [17,158], sliding mode control [159], Lyapunov-based nonlinear control [160] and model predictive control [157,161,162,163,164].

Multi-rotors are the most popular UAV type for many civilian applications due to their simplicity in mechanical design and control. Therefore, there have been many recent developments in nonlinear control of multi-rotors enabling high-speed navigation [59,77], aggressive flights [165,166,167] and aerial manipulation [168,169,170].

Quadrotor dynamics are differentially flat, which was shown in [68] (even under drag effects [153]). Differential-flatness denotes that all system variables (i.e., states and inputs) can be written in terms of a set of flat outputs (for example, [x,y,z,ψ]). That is, trajectories can be planned in the space of flat outputs, and it ensures that any smooth trajectory with proper bounded derivatives can be tracked. Hence, several control methods adopted a geometric-based control design utilizing the differential-flatness property such as [68,171]. Model predictive control was also considered in [164] where additional considerations, such as blade flapping and induced drag effects modeled as external disturbances, were included in the model and control design. Including such effects in the control design was considered by several other works such as [153,157,172]. Some other control designs for fixed-pitch multi-rotor UAVs were proposed; for example, see [19,159,162,173,174,175] and references therein.

Variable-pitch/omni-directional multi-rotors are fully actuated vehicles where translational and rotational degrees of freedom can be decoupled; examples of control methods developed for these vehicles can be found in [23,24,40]. Thus, these vehicles can even perform more complex tasks compared to fixed-pitch multi-rotors where controlling roll and pitch is essential to achieve required translations due to being underactuated systems. Control of single-rotor UAVs (helicopters) has also been tackled in several works using a similar cascaded structure. For example, a PID-based trajectory tracking controller was designed in [17], while a robust and perfect tracking (RPT) technique was suggested in [18].

Control of fixed-wing UAVs followed a similar control structure using decoupled control loops for translational and attitude dynamics. Control designs for fixed-wing UAVs take into consideration the models nonholonomic kinematic constraints, and many of the existing methods adopt path-following techniques based on guidance laws such as [158,160,176]. In [158], the control method adopted pure pursuit guidance and a decoupled proportional control for velocity and attitude. A similar control method was suggested in [160] based on LOS guidance algorithms and nonlinear control considering wind effects. Model predictive control was also considered in the path-following control design proposed in [161]. Alternatively, [154] presented control designs for fixed-wing UAVs based on linear pole placement and nonlinear structured multi-modal $H_{\infty }$ synthesis to track a reference air speed and flight path angle. Control of other UAV types has also attracted some interest in the community developing new control methods for hybrid UAVs [48,163], flapping-wing UAVs [33,36], etc.

## 5. Simultaneous Localization and Mapping (SLAM)

Localization is the process of determining the vehicle’s position with respect to a reference frame. This can be achieved given a certain map based on the newly obtained sensors information. On the contrary, mapping is the process of building a map representation of the environment given localization information. Thus, navigation in unknown environments requires both these processes to be carried out online simultaneously, which is known as simultaneous localization and mapping (SLAM). Development of SLAM methods is a very active field of research in robotics as the performance of map-based navigation methods relies on SLAM accuracy. This overview is not intended to provide a detailed survey of SLAM methods; however, the reader is referred to the following surveys for more details on recent developments in this area [177,178,179]. However, some of the recent state-of-the-art developments are briefly summarized in this section for the sake of completion.

Existing SLAM methods can be classified as either LiDAR-based or vision-based. LiDAR-based methods adopt scan matching algorithms, and they offer better accuracy (ex. see [180,181,182,183,184]). However, vision-based SLAM methods have become more popular for UAVs due to the lower cost and light weight of cameras compared to LiDARs. According to [178], these can be classified into feature-based [185,186,187], direct [188,189] or RGB-D camera-based methods [190,191]. Feature-based methods rely on detecting and extracting features from an input image to be used for localization which can be challenging in textureless environments. On the contrary, direct methods use the whole image directly, offering more robustness at the expense of increased computational cost. RGB-D camera-based methods combine both image and depth information in their formulation.

## 6. Summary of Recent Developments

Table 2 summarizes some of the recent contributions made towards developing fully autonomous UAVs, based on the surveyed works, in terms of control, perception, SLAM, motion planning and exploration capabilities.

## 7. Open-Source Projects

There have been many developments in the field of UAVs in terms of perception, control, SLAM and path planning over recent years. Implementing a complete autonomous navigation stack would require a large team with different skill sets in these areas or collaborations among research groups. Moreover, a lot of time needs to be invested in implementation and dealing with technical issues to ensure the reliability of the overall system. Open-source projects contributed by many researchers have made it possible for others in the community to focus on the development and improvement of a specific navigation component related to their research while easily integrating with other components made available by researchers, saving a lot of development time. Table 3 shows a list of some existing open-source projects and tools useful for autonomous UAV research and development.

## 8. Research Challenges

The navigation problem for UAVs remains a very challenging one due to the wide range of tasks they are needed for. Navigating in unknown and highly dynamic environments is one of the most challenging problems, especially for micro-UAVs with limited payload capacity and onboard computation capabilities. Some of the existing 3D reactive navigation approaches were developed based on conservative assumptions about obstacles. Similarly, map-based local trajectory planning methods tend to simplify the problem by relaxing the collision avoidance constraints to make the optimization problem more tractable in real-time. Overall, more theoretically well-founded, and computationally efficient navigation solutions are needed to provide a high level of safety guarantees.

Moreover, map-based navigation approaches are highly dependent on the performance of the localization system where a lot of ongoing research is focused on that area. Due to the limited payload capacity of small and micro-UAVs, vision sensors are the main source of information for localization and obstacle detection. However, this can be challenging in textureless environments. Furthermore, the small FOV provided by these sensors encourages more research in developing perception-aware navigation methods (ex. see [80,101,102,103,104,105]) to be able to maintain information about obstacles during avoidance maneuvers.

Developing fully autonomous UAVs targeting specific applications may involve additional layers of control; therefore, some of the applications where UAVs are used or can be potentially deployed are provided in the next section to bring to the reader’s attention the complexity and potential challenges in these areas. Moreover, using multiple UAVs in collaboration to carry out tasks can increase efficiency and reduce mission time. This makes the navigation and control problems of multi-vehicle UAVs an active field of research due to the higher complexity of these problems, which is highlighted in Section 8.2.

### 8.1. UAV Applications

As UAVs continue to emerge in new applications, new challenges arise based on the complexity of required tasks. New developments in technologies related to UAVs can also open research directions to develop new advanced navigation algorithms that would not have been possible with existing older technologies. Over the past decade, UAVs have been utilized in many applications. However, many of these applications still do not adopt fully autonomous solutions due to the involved operational risks and immaturity of research related to some of these particular applications. Thus, this section explores different areas where UAVs are currently used or needed to attract more interest in using UAVs and to guide further developments for UAV technologies supporting these applications with increased autonomy levels.

#### 8.1.1. Precision Agriculture

*Precision Agriculture (PA)* has attracted a lot of interest recently with a main goal of applying efficient solutions or resource management of soil and crops using different means of technology. UAVs offer great mobile solutions to increase productivity and to save resources in this area. Example applications related to PA include remote sensing [216,217,218], mapping [219,220], pests control [221,222], weed control [219,223,224,225] and harvesting [226].

Using UAVs for remote sensing applications in PA provides inspection data at higher temporal and spatial resolution than satellite imagery [227]. UAVs can be equipped with different sensors to provide rich information about soil condition, crop growth and plants biomass and vigor. For example, thermal and RGB images collected by a UAV can help farmers identify crop water stress. Similarly, images collected from multi-spectral and hyperspectral cameras can be used to determine vegetation indices, which is a good way for continuous monitoring of crop variability and stress conditions [228]. These cameras are very expensive compared to thermal and RGB cameras, which can be a limiting factor in some cases.

In remote sensing applications, coverage path planning algorithms are normally applied generating optimal paths (ex. back-and-forth motion patterns) to survey areas of interest. For high-altitude flights, the coverage path planning problem can be simply solved using classical approaches assuming an obstacle-free flight space. In this case, simple path following control can be applied. This problem becomes more complex on the high level coverage planning when considering no-flight zones (i.e., obstacles), multi-UAV cases and low-altitude flights. These cases also require proper local motion planning to handle dynamic obstacles, such as people and other noncooperative UAVs, and static obstacles such as trees, buildings, etc. Moreover, advanced control methods are needed to perform autonomous tasks such as harvesting, irrigating and weed control.

#### 8.1.2. Search and Rescue

Search and Rescue (SAR) operations have evolved over the years in considering robotic aid for improved results. This attracted attention to the field of Disaster Robotics. UAVs can add great value to SAR missions by carrying out different tasks including: localizing and tracking victims; survivors’ situation and environment assessments; delivering aid kits such as first aid and self-inflating emergency flotation devices; communicating messages from rescue teams to victims; providing wireless communication networks between SAR teams in remote inaccessible areas [229]. Clearly, the system will vary depending on the delegated task. For example, UAVs equipped with color and/or thermal cameras are used to localize victims either manually by providing visual feedback to a remote operator or autonomously using computer vision algorithms with proper onboard GPU power. On the other hand, UAVs used to deliver aid kits need to have higher payload and aerial manipulation capability. Providing wireless communication networks can be valuable in marine SAR missions where it is hard to set up ground networks.

Developing fully autonomous SAR-enabled UAVs requires suitable navigation strategies depending on the environment (indoors or outdoors) and the required motion objectives. For example, some cases require UAVs to survey areas of concern where the application of coverage motion planning algorithms is needed similar to remote sensing applications. 3D exploration techniques can also be applied in indoors environments which is still an active research problem. Navigation in harsh indoor environments, such as tunnels and collapsed buildings, adds more challenges towards achieving fully autonomous operations. For example, there is a need for suitable control methods to handle flights in confined spaces, well developed SLAM methods to deal with poor conditions, and proper perception and obstacle avoidance capabilities. Solutions based on the use of cooperative UAVs in SAR missions are also attractive but require further development due to the higher complexity of these solutions.

Examples of UAV-based solutions for SAR applications in different environments and scenarios such as: remote disaster areas and wilderness SAR [1,230,231,232]; urban SAR (ex. collapsing buildings) [233,234,235]; underground tunnels [236,237]; and marine SAR [238,239,240,241].

#### 8.1.3. Animal Control and Wildlife Monitoring

Another area where UAVs can be very useful is livestock and wildlife monitoring. For example, UAVs can be used to count, classify, and track livestock animals [242,243] to optimize hunting and harvesting in farms. To achieve these tasks, UAVs need to be equipped with proper sensors such as RGB or thermal cameras. Monitoring wildlife is also important to manage the population of threatened and invasive species (see [244,245,246]). Moreover, UAVs have also been considered for detecting and tracking some marine wildlife swimming close to the surface such as hammerhead sharks [247]. Depending on the environment and UAV sensors configuration, perception-aware trajectory planning methods can be applied in the case of animal tracking to make sure that the targeted animal remains within its field of view. Another interesting application is the use of UAVs for herding of birds [248] and farm animals [249]. In many of these applications, the UAV is usually flying at a higher altitude than the targets, which makes it valid to assume that the flight space is less crowded with obstacles. However, complete autonomous solutions for herding require more development for motion planning as the problem relies on the dynamical behavior of the animals. For example, motion planning can be combined with prediction methods to predict animals’ future trajectories. However, it is important to consider some challenging factors in these applications such as the effect of UAV sound on wildlife under study [250] and other general effects which requires a code of practice for the use of UAVs in biological field research [251].

#### 8.1.4. Weather Forecast

The low cost of UAVs makes them good tools to collect more information about hurricanes and tornadoes using dedicated sensors. Such collected data can help scientists to build better understanding of the trajectories of hurricanes and tornadoes. UAVs can also provide advanced warning and damage assessment for thunderstorms and tornadoes. There are few works in this area such as [252,253,254,255].

#### 8.1.5. Construction

In the construction domain, deployment of UAVs in construction sites is good for aiding high-level management. Applications of UAVs in construction include [256]: building inspection; post-disaster damage assessment; site surveying and mapping; safety inspection; and progress monitoring. For example, see [256,257,258,259,260] and references therein.

Navigating in construction sites can be challenging due to being highly dynamic and crowded environments. Some tasks may also require UAVs to fly very close to buildings such as facade inspection and scanning buildings to build 3D models. This can be a potential application for reactive methods to maintain a certain distance from the building.

#### 8.1.6. Oil and Gas

UAVs have also started to attract interest in the oil and gas industry. For example, UAV-based magnetic surveys can be used to detect and identify abandoned wells [261]. The use of UAVs for monitoring and inspection of oil and gas pipeline networks can aid in preventing failures, detecting problems over time and performing repair activities [262,263,264]. Another possible application is the detection of gas leaks where UAVs with in situ sensors can be used [265].

#### 8.1.7. Other

Other UAV applications include: solar panel inspection [266,267]; power line and tower inspections [268,269]; water quality monitoring [270]; magnetic field mapping [271]; load transportation [272,273]; contact inspection tasks [274]; road safety and traffic monitoring [275,276,277]; aerial photography and cinematography [278]; entertainment and aerial shows [279,280,281,282]; firefighting [283,284,285].

### 8.2. Multi-UAV and Networked Systems

The use of multi-UAV systems has become more desirable in many applications for improved performance; thus, developing these systems is currently a very active field of research. Various challenges arise in this area to develop autonomous UAV swarms in tasks assignments, communication, trajectory planning and coordinated control. Different interactions between the vehicles are needed in order to collaboratively achieve a global objective assigned to the group.

Algorithms developed for Multi-UAV systems can be either *centralized*, *decentralized* or *distributed*. *Centralized* approaches are implemented on a central computer where trajectories are computed for all agents/vehicles within the system. This requires measurements from all agents to be available to the central computer. Centralized algorithms can produce globally optimal solutions; however, the overall system is prone to failure if the central computer fails, or a communication problem occurs. On the other hand, *decentralized* and *distributed* algorithms offer more robustness and scalability with a more computationally efficient solution to systems with large number of vehicles. These approaches enable each vehicle to compute their own trajectories and control actions based on local interactions with neighboring UAVs either by relying directly on its sensors’ measurements (*decentralized*) or by combining these with information communicated by neighbor vehicles (*distributed*). In other words, distributed methods distribute the computation and communication load among the vehicles [286] to collaboratively serve a global group objective.

Formation control is one of the challenging areas for multi-UAV systems where each vehicle has a specific role with some constraints on its states [287] to achieve global group objective(s). The common formation control structures in the literature are *leader-follower*, *virtual* and *behavioral-based structures* [288]. A physical vehicle is set to be followed by the remaining vehicles within the system in a certain manner in *leader-follower* structures such as [289,290,291,292,293,294,295]. On the contrary, approaches with *virtual structure* achieves motion formation through forcing each vehicle to follow a corresponding virtual target (or reference trajectory) such that the selection of these virtual references contributes towards the global objective; for example, see [296,297,298,299,300,301]. *Behavioral-based* structure comprises of a set of rules followed by the vehicles contributing towards the collective behavior; flocking control, based on artificial potential fields, belongs to this category where a group of interacting agents move together to achieve some global objectives. One of the early models capturing the local interactions between agents under a flocking behavior is Reynolds’ model for the aggregate motion of flocks, which is based on three main rules: flock centering (cohesion), collision avoidance (separation) and velocity matching (alignment) [302,303]. Several works have addressed the flocking problem such as [303,304,305,306,307,308,309,310,311]. Different simplifications are normally made to deal with the high dimensionality of this problem, which includes considering simpler motion models such as single integrator [309,312,313], double integrator [303,304,311,314], nonholonomic models [305,306,307,308,315] and Euler–Lagrangian systems [310,316].

Many works have tackled the 3D motion coordination and collision avoidance problem for UAV swarms through a hierarchical approach with trajectory planning formulated as an optimization problem, similar to what was discussed earlier for single-UAV systems. Different centralized, decentralized and distributed approaches can be seen in the literature considering homogeneous and heterogeneous multi-UAV systems in addition to considering multi-vehicle systems working with aerial and ground vehicles; for example, see [79,317,318,319,320,321,322,323,324,325,326,327].

An example task that can be carried out by UAV swarms is carrying and transporting objects individually [168,328,329,330] or collaboratively [295,331,332,333,334,335]. Other applications where multi-UAV systems were deployed include distributed target search and tracking [336,337,338,339], distributed monitoring and surveillance [340,341,342,343] and cooperative mapping [344,345]. The research on cooperative mapping in unknown environments, or swarm SLAM, is is not mature yet with not enough established methodologies according to [346], which motivates more research in this promising area.

It is also very important to keep in mind communication challenges when designing control methods for large-scale networked multi-vehicle systems. Application of Networked Control Systems (NCSs) theory is thus suitable when analyzing the operation of networks of collaborating autonomous UAVs [347,348,349,350,351,352]. This can help considering additional practical aspects of NCSs in the overall system design such as delays introduced in communication channels [347,353,354], noises [355,356], loss/corruption of data [353,354] and bandwidth constraints [357,358,359,360].

## 9. Conclusions

Rapid advances in UAV-related technologies allowed great development towards achieving fully autonomous operations in the areas of control, motion planning, perception and localization and mapping. This paper presented a survey about some recent advancements in these areas focusing more on allowing advanced autonomous 3D collision-free navigation for UAVs. The main differences between the adopted motion planning algorithms and control strategies were also highlighted, showing the advantages and disadvantages between different methods. This can provide guidance for researchers to determine suitable navigation methods based on their specific applications. Moreover, a list of some existing open-source projects was provided to aid researchers in quickly developing and deploying innovative technologies for UAVs. Developing fully autonomous UAVs remains very challenging due to the wide range of new applications and the various levels of the tasks’ complexity. Hence, active research challenges were also highlighted in this paper, including recent developments in motion control for multi-UAV systems. Additionally, several applications were mentioned to encourage the development of more mature fully autonomous solutions dedicated to emerging UAV applications.

## Figures and Tables

**Table 1:** A summary of surveyed local motion planning methods for UAVs.

Refs.	Control Structure	Local Motion Planning	Model	Dynamic Environment
[55]	I/II	sampling-based path planning	2D Kinematics (nonholonomic)	✓
[56]	I/II	sampling-based path planning	3D Single-rotor Dynamics	
[57]	I/II	sampling-based path planning	3D Kinematics (nonholonomic)	✓
[58]	I/II	sampling-based path planning	3D Kinematics (holonomic)	
[60]	I/II	graph-based path planning	3D Kinematics (holonomic)	✓
[61,63]	I/II	optimization-based path planning	3D Kinematics	
[62]	I/II	optimization-based path planning	3D Quadrotor Dynamics	
[59,68,70,72,73,88]	II/III	optimization-based trajectory generation using QP with corridor-like constraints	3D Quadrotor Dynamics	
[78,82,93]	III	optimization-based trajectory planning using QP	3D Dynamics (acceleration/jerk input)	
[69,85]	III	optimization-based trajectory planning using unconstrained QP	3D Quadrotor Dynamics	
[71,74,75]	III	optimization-based trajectory planning with obstacles constraints	3D Quadrotor Dynamics	
[77,81,90,92,101]	III	motion primitives	3D Quadrotor Dynamics	
[94]	III	motion primitives	3D Kinematics (holonomic)	
[91]	III	motion primitives	3D Kinematics (nonholonomic)	
[79,80]	III	perception-aware trajectory planning	3D Dynamics (jerk input)	✓
[101,102,103,104,105]	III	perception-aware trajectory planning	3D Quadrotor Dynamics	
[96,100]	III/IV	nonconvex optimization with obstacles constraints using NMPC	3D Quadrotor Dynamics	✓
[83,84,86]	III/IV	mapless vision-based trajectory planning using depth images	3D Dynamics (jerk input)	
[115,116,117,118,119,120]	IV	Geometric-based (collision cones) reactive control	3D Kinematics	✓
[125,126]	IV	reactive control based on Velocity Obstacle (VO)	3D Kinematics	✓
[127,128,129]	IV	reactive control based on artificial potential field	3D Kinematics (nonholonomic)/Quadrotor Dynamics	✓
[121]	IV	nature-inspired reactive control	3D Kinematics (nonholonomic)	
[134]	IV	vision-based reactive control	2D Kinematics	
[131,132,133]	IV	real-time path deformation (reactive)	3D Quadrotor Dynamics	✓
[135]	IV	vision-based reactive control based on NMPC	3D Quadrotor Dynamics	✓
[136,137,138,139,140,141]	IV	deep reinforcement learning	2D Kinematics	
[142]	IV	deep reinforcement learning	2D Kinematics	✓
[143]	IV	deep reinforcement learning	3D Kinematics	
[144,145,146,147,148]	IV	deep neural networks	2D Kinematics	
[149,150]	IV	deep neural networks	3D Kinematics	
[151]	IV	deep neural networks	3D Kinematics	✓

**Table 2:** A summary of some recent developments for UAVs in control, perception, SLAM and motion planning.

References	Control	Perception	SLAM	Motion Planning	Exploration
[192]		✓			
[55,57,60,61,62,63,70,71,72,73,75,77,78,79,80,81,82,83,84,85,86,90,92,93,94,96,101,104,115,117,118,119,120,121,125,126,127,128,130,132,136,137,138,139,140,141,142,143,144,145,146,147,148]				✓	
[59,71,134,149,150,151,193]		✓		✓	
[58,194,195,196]				✓	✓
[17,18,19,48,153,154,158,159,160,161,162,163,164,171,173,176]	✓				
[178,180,181,182,183,184,185,186,187,188,189,190,191]			✓		
[56,68,69,74,91,102,103,105,129,131,135]	✓			✓	
[100]	✓	✓		✓	
[197]	✓	✓	✓		
[88,198,199]	✓	✓	✓	✓	
[200]	✓	✓	✓	✓	✓

**Table 3:** Open-source projects and tools for UAV development.

	Name	Description	Source
Navigation Stack	Vision-based navigation for MAVs [201]	provides an open-source system for MAVs based on vision-based sensors including control, sensor fusion, mapping, local and global planning	http://github.com/ethz-asl/voxblox http://github.com/ethz-asl/rovio http://github.com/ethz-asl/ethzasl_msf http://github.com/ethz-asl/odom_predictor http://github.com/ethz-asl/maplab http://github.com/ethz-asl/mav_control_rw
	PULP-DroNet [202]	a deep learning-powered visual navigation engine for nano-UAVs	https://github.com/pulp-platform/pulp-dronet
LiDAR-based SLAM	Google’s Cartographer [181]	provides a real-time SLAM solution in 2D and 3D	https://github.com/cartographer-project/cartographer
	hdl_graph_slam [182]	a real-time 6DOF SLAM using a 3D LIDAR	https://github.com/koide3/hdl_graph_slam
	loam_velodyne [180]	Laser Odometry and Mapping	https://github.com/laboshinl/loam_velodyne
	A-LOAM	Advanced implementation of LOAM	https://github.com/HKUST-Aerial-Robotics/A-LOAM
	FLOAM	a faster and optimized version of A-LOAM and LOAM	https://github.com/wh200720041/floam
Vision-based SLAM	ORB SLAM [186]	a keyframe and feature-based Monocular SLAM	https://openslam-org.github.io/orbslam.html
	ORB SLAM 2 [187]	a real-time SLAM library for Monocular, Stereo and RGB-D cameras	https://github.com/raulmur/ORB_SLAM2
	LSD-SLAM [188]	a Large-Scale Direct Monocular SLAM system	https://github.com/tum-vision/lsd_slam
	SVO Semi-direct Visual Odometry [203]	a semi-direct monocular visual SLAM	https://github.com/uzh-rpg/rpg_svo
	PTAM [185]	a monocular SLAM system	https://github.com/Oxford-PTAM/PTAM-GPL
	RTAB-Map [204,205]	RGB-D, Stereo and Lidar Graph-Based SLAM algorithm	http://introlab.github.io/rtabmap
	ElasticFusion [206]	Real-time dense visual SLAM system using RGB-D cameras	https://github.com/mp3guy/ElasticFusion
	Kintinuous [190]	Real-time dense visual SLAM system using RGB-D cameras	https://github.com/mp3guy/Kintinuous
Motion Planning	Fast-Planner [87]	a set of planning algorithms for fast flights with quadrotors in complex unknown environments	https://github.com/HKUST-Aerial-Robotics/Fast-Planner
	FUEL [207]	a hierarchical framework for Fast UAV Exploration	https://github.com/HKUST-Aerial-Robotics/FUEL
	EGO-Planner	Gradient-based Local Planner for Quadrotors	https://github.com/ZJU-FAST-Lab/ego-planner
	TopoTraj [208]	a robust planner for quadrotor trajectory replanning based on gradient-based trajectory optimization	https://github.com/HKUST-Aerial-Robotics/TopoTraj
	toppra [209]	a library for computing time-optimal trajectories subject to kinematic and dynamic constraints	https://github.com/hungpham2511/toppra
	Open Motion Planning Library	a library for sampling-based motion planning algorithms	https://ompl.kavrakilab.org/core/index.html
	AIKIDO	a C++ library for motion planning and decision making problems	https://github.com/personalrobotics/aikido
	PathPlanning	a collection of search-based and sampling-based path planners implemented in Python	https://github.com/zhm-real/PathPlanning
Control	mav_control_rw [164]	Linear and nonlinear MPC controllers for Micro Aerial Vehicles	https://github.com/ethz-asl/mav_control_rw
	rpg_mpc [102]	Perception-Aware MPC for quadrotors	https://github.com/uzh-rpg/rpg_mpc
	ACADO Toolkit	collection of algorithms for automatic control and dynamic optimization	http://acado.github.io/
	Control Toolbox	a C++ library for robotics addressing control, estimation and motion planing	https://github.com/ethz-adrl/control-toolbox
	PX4	an open-source flight control software for UAVs	https://px4.io/
	ArduPilot	an open-source flight control software for UAVs	https://ardupilot.org/
Perception	Augmented Autoencoders [210]	3D object detection pipeline from RGB images	https://github.com/DLR-RM/AugmentedAutoencoder
	MoreFusion [211]	a perception pipeline for 6D pose estimations of multi-objects	https://github.com/wkentaro/morefusion
	OpenCV	an optimized computer vision library	https://opencv.org/
	Point Cloud Library (PCL)	efficient point cloud processing C++ library	https://pointclouds.org/
	cilantro [212]	efficient point cloud processing C++ library	https://github.com/kzampog/cilantro
Simulators	Gazebo	a robot simulator	http://gazebosim.org/
	CoppeliaSim/V-REP	a robot simulator	https://www.coppeliarobotics.com/
	Webots	a robot simulator	https://cyberbotics.com/
	Hector Quadrotor	provides simulation tools for quadrotors (ROS-based)	http://wiki.ros.org/hector_quadrotor
	RotorS [213]	a set of tools to simulate multi-rotors in Gazebo	https://github.com/ethz-asl/rotors_simulator
General	Robot Operating System (ROS)	a middleware to facilitate building large robotic applications	https://www.ros.org/
	Ceres Solver	a C++ library for solving large optimization problems	http://ceres-solver.org/
	g2o	a C++ framework for graph-based nonlinear optimization	https://github.com/RainerKuemmerle/g2o
	NLopt	a nonlinear optimization library	https://nlopt.readthedocs.io/en/latest/
	Optimization Engine (OpEn)	a fast solver for optimization problems in robotics	https://nlopt.readthedocs.io/en/latest/
	Interior Point OPTimizer (Ipopt) [214]	a software and library for solving large-scale nonlinear optimization problems	https://github.com/coin-or/Ipopt
	SNOPT	an optimizer for large-scale nonlinear optimization problems	https://ampl.com/products/solvers/solvers-we-sell/snopt
	ifopt [215]	a light-weight C++ interface to Nonlinear Programming Solvers Ipopt and Snopt	https://github.com/ethz-adrl/ifopt
